# Lung transcriptomics reveals the underlying mechanism by which aerobic training enhances pulmonary function in chronic obstructive pulmonary disease

**DOI:** 10.1186/s12890-024-02967-1

**Published:** 2024-03-26

**Authors:** Jian Li, Cai-tao Chen, Peijun Li, Xiaoyun Zhang, Xiaodan Liu, Weibing Wu, Wei Gu

**Affiliations:** 1 Department of Traditional Chinese Medicine, Changhai Hospital, Naval Medical University (Second Military Medical University), 200433 Shanghai, PR China; 2 Department of Sports Rehabilitation, Shanghai University of Sport, No. 399 Changhai Road, Yangpu District, 200438 Shanghai, PR China; 3 Department of Rehabilitation Medicine, Shanghai Fourth People’s Hospital, School of Medicine, Tongji University, 200434 Shanghai, PR China; 4 School of Rehabilitation Science, Shanghai University of Traditional Chinese Medicine, 201203 Shanghai, PR China; 5 Laboratory Department of the 908th Hospital of the Joint Logistics Support Force, 330001 Nanchang, PR China; 6 Faculty of Traditional Chinese Medicine, Naval Medical University (Second Military Medical University), No. 800 Xiangyin Road, Yangpu District, 200433 Shanghai, PR China

**Keywords:** Exercise training, Chronic obstructive pulmonary disease, Transcriptomics, Pulmonary function, Rehabilitation

## Abstract

**Background:**

Aerobic training is the primary method of rehabilitation for improving respiratory function in patients with chronic obstructive pulmonary disease (COPD) in remission. However, the mechanism underlying this improvement is not yet fully understood. The use of transcriptomics in rehabilitation medicine offers a promising strategy for uncovering the ways in which exercise training improves respiratory dysfunction in COPD patients. In this study, lung tissue was analyzed using transcriptomics to investigate the relationship between exercise and lung changes.

**Methods:**

Mice were exposed to cigarette smoke for 24 weeks, followed by nine weeks of moderate-intensity treadmill exercise, with a control group for comparison. Pulmonary function and structure were assessed at the end of the intervention and RNA sequencing was performed on the lung tissue.

**Results:**

Exercise training was found to improve airway resistance and lung ventilation indices in individuals exposed to cigarette smoke. However, the effect of this treatment on damaged alveoli was weak. The pair-to-pair comparison revealed numerous differentially expressed genes, that were closely linked to inflammation and metabolism.

**Conclusions:**

Further research is necessary to confirm the cause-and-effect relationship between the identified biomarkers and the improvement in pulmonary function, as this was not examined in the present study.

## Background

Chronic obstructive pulmonary disease (COPD) is a prevalent and preventable respiratory condition characterized by persistent symptoms and restricted airflow, which is primarily caused by abnormalities in the respiratory tract and/or alveolus, often resulting from high exposure to harmful particles or gases like cigarette smoke, and host factors, including abnormal lung development [1–3]. Recently, attention has been paid to the extrapulmonary effects of COPD, given the impact that severe comorbidities may impact morbidity and mortality [4]. COPD has emerged as the third leading cause of death worldwide [5], affecting approximately 10.1% of the global population [6] and resulting in five million fatalities each year. The incidence rate of COPD among individuals over 40 years old is alarmingly high, reaching 13.7% in China [7], and this number continues to increase due to various factors, such as air pollution and smoking. The high incidence and fatality rates of COPD indicate the need for effective therapies to prevent disease progression and reduce severity. However, airway management during disease remission has not received adequate attention compared to intervention during acute exacerbation of COPD. This lack of attention is one of the factors that negatively impacts the quality of life of patients with COPD [8]. As a result, current pulmonary rehabilitation research is focused on exploring airway management during the remission period of COPD.

Aerobic training and resistance exercise are the primary methods of pulmonary rehabilitation for COPD patients in remission. These interventions have been found to significantly improve pulmonary ventilation status [9, 10]. As a classic pulmonary rehabilitation training method, low-moderate intensity aerobic training is highly recommended for airway management during the remission period of COPD. Clinical studies have shown that aerobic exercise training for a period of four weeks improves respiratory status [11], fatigue, maximum exercise capacity, and quality of life in patients suffering from COPD [12]. This improvement has been observed even in patients in advanced stages of the disease [13]. Additionally, short-term training after an acute exacerbation has been shown to reduce the frequency of exacerbations and lengthen readmission time in patients with COPD [14]. Animal models have also demonstrated the positive effects of exercise training. In mice exposed to cigarette smoke, regular exercise training mitigated lung damage by reducing the mean alveolar intercept and superoxide production [15]. Moreover, a previous study confirmed that exercise protected against lung injury caused by cigarette smoke exposure (CSE), and this effect was attributed to the role of anti-inflammatory mediators and antioxidant enzymes in the development of COPD after exercise adaptation [16]. Studies have also confirmed that exercise reduces signal transducer and activator of transcription 3 (STAT3) levels [17] and upregulates antioxidant genes [18], such as nuclear factor erythroid 2 (NRF2) and heme oxygenase 1 (HMOX1). Although numerous studies have investigated the mechanism of exercise-mediated lung rehabilitation, this issue remains complex and unclear. Exercise training, with its multitarget stimulatory approach, may contribute to the rehabilitation of respiratory function in COPD through various therapeutic targets. As such, identifying biomarkers of bodily changes following exercise training could help elucidate the intricate mechanisms of exercise rehabilitation.

Transcriptomics is an emerging discipline that has become an important method for biomarker research. By simultaneously detecting thousands of RNAs, RNA-sequencing, a transcriptome analysis method, accurately and efficiently identifies the overall transcriptional activity of cells or tissues using deep sequencing technology [19]. Numerous differentially expressed genes (DEGs) were identified according to the mRNA profile, which provided a basis for revealing the pathogenesis and rehabilitation-related mechanism of COPD. Currently, various genes and molecular signaling pathways associated with COPD have been identified through transcriptomics, but the results of different studies are not highly reproducible and the exploration of exercise rehabilitation-related mechanisms is still in its infancy. Therefore, confirmed signaling pathways and biomarkers can be investigated through transcriptomic analysis to explore the underlying mechanism of exercise training-mediated rehabilitation of respiratory function in COPD.

In this study, a COPD model was established through CSE, and a 9-week exercise intervention was administered. The rehabilitative effect of exercise training on the pulmonary function of mice with COPD was then determined, followed by mRNA sequence analysis of the lung tissues of three groups of mice. The biological functions of DEGs and their enriched pathways were screened through Gene Ontology (GO) functional analysis and Kyoto Encyclopedia of Genes and Genomes (KEGG) analysis, revealing potential therapeutic mechanisms for exercise in ameliorating pulmonary dysfunction in COPD.

## Methods

### Animals, smoke exposure and exercise protocol

Eight-week-old male C57BL/6J mice, purchased from Charles River Laboratories (Pinghu, China), were housed in cages with free access to food and tap water under standard conditions of humidity (60 ± 10%), temperature (21 ± 2 ℃), and light (12 h light/12 h dark cycle). Mice were randomly divided into three groups (8 mice in each group): the **control group (no training + air, CG), model group (no training + smoke, MG) and exercise group (treadmill training + smoke, EG)**. The methods were carried out in accordance with the approved guidelines of the Experimental Animal Care and Ethics Committee of the Shanghai University of Traditional Chinese Medicine (No. PZSHUTCM210312012).

The mice received whole-body CSE for 24 weeks to establish a COPD model, and the CSE protocol was described in a previous study [20]. Briefly, an increasing number (up to 20) of commercially filtered cigarettes (10 mg tar, 0.9 mg nicotine and 12 mg carbon monoxide per cigarette) were lit and passed through a passive smoke exposure system (PAB-S200; Beijing Bestlab High-Tech Co., Ltd. Beijing, China) to produce cigarette smoke. Animals in the MG and EG groups were exposed to smoke for 1 h twice a day, 6 days a week. During the modeling phase, CG mice were exposed to air in the same environment.

**Fig. 1:**
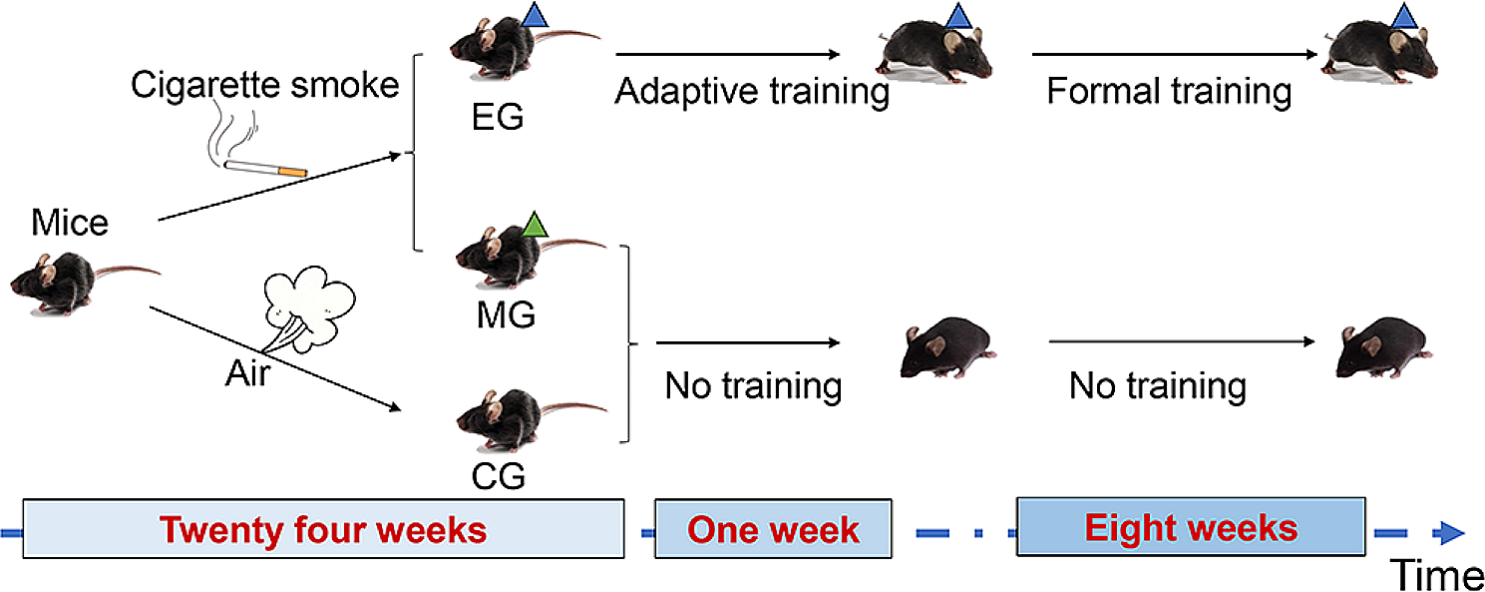
COPD mouse model establishment process and exercise intervention protocol. The mice were randomly divided into CG, MG and EG (*n* = 8). MG and EG were exposed to smoke for 24 weeks to replicate the COPD model, and the latter received 9 weeks of aerobic exercise intervention. Finally, multiple indicators in each group were examined. CG, control group; MG, model group; EG, exercise group. Values represented the means ± SD

After the establishment of the COPD model, EG mice underwent adaptive treadmill training for one week. The maximum exercise capacity was tested as follows: warm-up was performed at 5 m/min; 5 min later, the running speed was gradually increased (2 m/min every 3 min) until the mice were exhausted, and the running speed was recorded as the maximum exercise capacity of the mice. In the follow-up 8-week exercise intervention, a treadmill running intervention was carried out with an appropriate exercise intensity of moderate intensity (55% of the maximum exercise capacity) [21], for 60 min per day, six days a week. Throughout one week of adaptive training and eight weeks of formal training, the CG and MG mice were allowed to freely move in the cages without any artificial exercise intervention that the EG mice were exposed to the experimental protocol showed in Fig. 1.

### Pulmonary function

Mice were anesthetized by intraperitoneal injection with tribromoethanol sodium at a dose of 350 mg/kg. Once the righting reflex and pain stimulation response disappeared, the mice were prepared for dissection. The neck skin was cut lengthwise and the subcutaneous tissue and neck muscles were carefully separated to expose and dissociate the trachea. The trachea was cut horizontally using tissue scissors between the tracheal cartilage rings, and a tracheal cannula was inserted. The pulmonary function of mice was assessed using the small animal Pulmonary Function Test System (PFT, DSI, St Paul, USA).

### HE staining

The mice were sacrificed using decapitation after cardiac arrest and the disappearance of nerve reflexes were confirmed. The lung tissue was carefully removed under atmospheric pressure and the lung tissues of each group were subjected to the same pressure. Subsequently, the tissue was rinsed with normal saline and internally fixed using 4% paraformaldehyde. The following day, the tissue was embedded in wax blocks. It is worth noting that the lungs were not irrigated during this procedure to avoid causing any artificial damage to the lung tissue structure. Moreover, special care was taken to handle the lung tissue gently while clipping, so as to minimize any potential impact of human factors on the alveolar structure. Finally, the lung tissue was observed for morphological changes using HE staining. For each group, three sections were randomly chosen and three visual fields were randomly observed. The alveolar cross-sectional area was determined using the Image-Pro Express Image analysis system (Media Cybernetics, Maryland, USA).

### RNA sequence analysis and data processing

The GenSeq® rRNA Removal Kit (GenSeq, Inc., Shanghai, China) was utilized to eliminate ribosomal RNA (rRNA) from the samples, following the supplier’s instructions. After the removal of rRNA, the sequencing library was constructed using the specified procedure. Quality control and quantification of the constructed sequencing library were performed using the BioAnalyzer 2100 system (Agilent Technologies, Palo Alto, USA). The Illumina NovaSeq 6000 instrument (Illumina, Santiago, USA) was used for 150 bp double-ended sequencing. The Q30 value was utilized for raw data quality control. Cutadapt [22] (v1.9.3) was employed to remove low-quality reads and obtain high-quality clean reads. These clean reads were then aligned to the reference genome using HISAT2 [23] (v2.0.4). Next, HTSeq [24] (v0.9.1) was used to determine the original count number. Standardization was performed using edgeR [25], and multiple changes and P values between the two groups of samples were calculated to identify DEGs. GO functional and KEGG pathway analyses were performed using differentially expressed mRNAs.

For validation data, public genomics data in the Sequence Read Archive (SRA) database were downloaded. The dataset PRJNA976479 was used, and differential expression was assessed.

### Statistical analysis

Data were analyzed using Statistical Package for the Social Sciences software, version 25.0 (SPSS, Inc., Chicago, IL, USA). The distribution of pulmonary function and alveolar cross-sectional area data were tested using Kolmogorov‑Smirnov and Levene tests to ensure normality and variance homogeneity. Normally distributed data were presented as the mean ± standard deviation (SD), while skewed data are presented as the median (quartile range). One-way analysis of variance was used to compare the groups and the least significant difference method was used for further comparison. If the variances were inconsistent, Dunnett’s T3 test was used for multiple comparisons between groups. A statistically significant difference was considered present when the value of P was less than 0.05.

## Results

### Exercise training improved the pulmonary function of mice with COPD

**Fig. 2:**
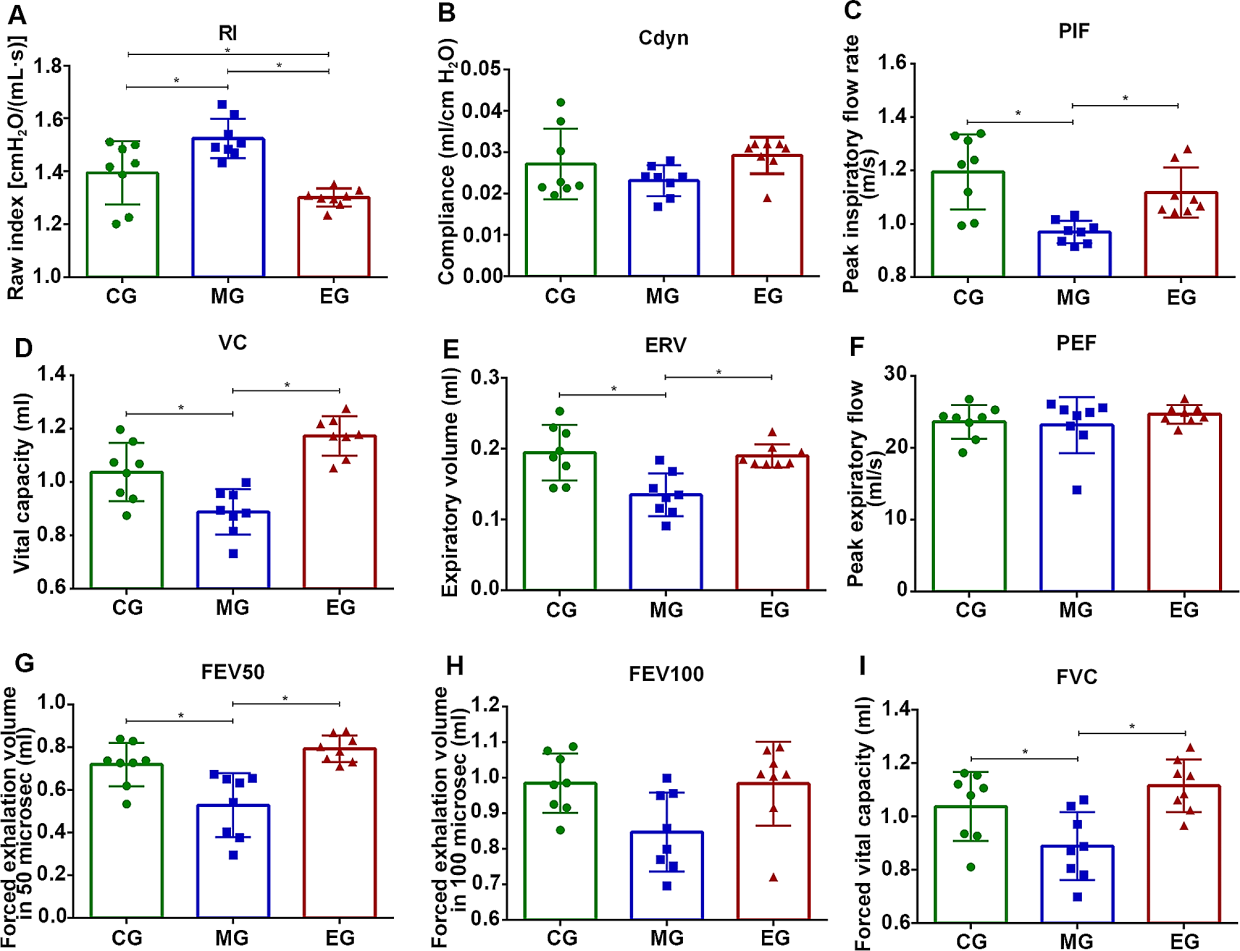
Exercise training improved the pulmonary function of COPD mice. Raw index (RI) (**A**), dynamic lung compliance (Cdyn) (**B**), peak inspiratory flow rate (PIF) (**C**), vital capacity (VC) (**D**), expiratory volume (ERV) (**E**), peak expiratory flow (PEF) (**F**), forced exhalation volume (FEV) in 50 microseconds (**G**), 100 microseconds (**H**) and forced vital capacity (FVC) (**I**) in three groups were showed orderly. Treadmill training decreased RI and increased PIF, VC, ERV, FEV50 and FVC in COPD mice. CG, control group; MG, model group; EG, exercise group. Values represented the means ± SD. ^*^P < 0.05

We found that CSE worsened airway resistance, but exercise intervention helped to reduce this effect **(**Fig. 2**)**. The raw value of airway resistance in the mice exposed to CSE was significantly higher than that in the control mice, but the mice in the exercise group had lower airway resistance than those in the CSE group. However, there was no significant difference in dynamic lung compliance (Cdyn) values among the three groups. The lung capacity indices of the MG mice exhibited a notable decrease in vital capacity (VC) and expiratory reserve volume (ERV) values. Nevertheless, after the intervention, the ventilation indices of the mice with COPD, VC and ERV, were significantly increased (*P* < 0.05). Additionally, CSE weakened the ventilation function of the mice, leading to significant decreases in forced vital capacity (FVC), forced expiratory volume in fifty milliseconds (FEV50) and peak inspiratory flow rate (PIF) values in the MG mice compared to the healthy mice (*P* < 0.05). Exercise intervention could improve these indices of ventilation function (*P* < 0.05).

### The limited positive impact of exercise training on lung structure

**Fig. 3:**
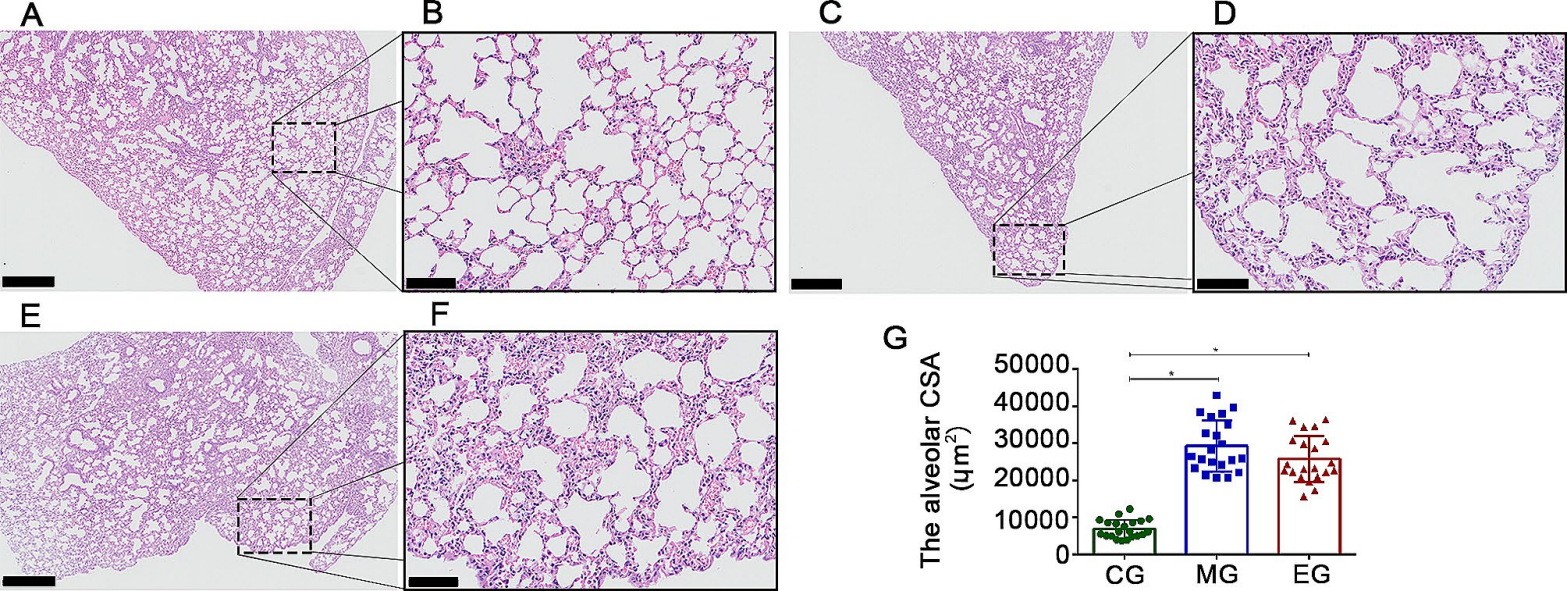
Effect of exercise training on lung structure in mice. The lung tissue structure of mice in blank group (**A**, bar = 500 μm; **B**, bar = 100 μm), model group (**C**, bar = 500 μm; **D**, bar = 100 μm) and exercise group (**E**, bar = 500 μm; **F**, bar = 100 μm) was observed, and the alveolar cross-sectional area of mice were compared in three randomly selected fields (**G**). The lung structures of MG and EG mice exhibited typical emphysema, compared to healthy mice. After exercise intervention, the alveolar size of EG mice did not show significant changes. CG, control group; MG, model group; EG, exercise group; CSA, cross-sectional area. Values represented the means ± SD. ^*^P < 0.05

The lung tissue of the mice exposed to ambient air displayed thin alveolar walls and normally sized alveoli. However, the lung tissue of the mice exposed to cigarette smoke exhibited destruction of the alveolar septum, enlarged alveoli, the presence of inflammatory cells, and even thickened interstitial tissue in certain visual fields. Notably, airway histological findings of the mice who exercised showed similar features to those in the MG group. Further comparison of randomly selected visual field samples showed a significant increase in alveolar cross-sectional area in the MG mice, indicating that exercise intervention did not prevent alveolar fusion and expansion **(**Fig. 3**)**.

### DEGs in pairwise comparisons

**Fig. 4:**
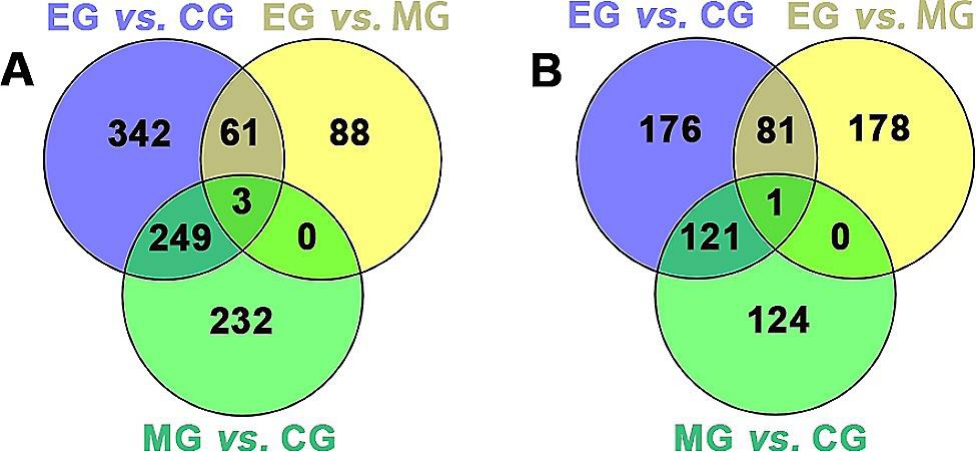
Venn diagrams of DEGs were identified in different comparisons, including (**A**) upregulated DEGs and (**B**) downregulated DEGs. The venn diagrams revealed overlapping DEGs in the comparisons between groups, including three upregulated DEGs and one downregulated DEGs in the three comparisons. CG, control group; MG, model group; EG, exercise group

**Fig. 5:**
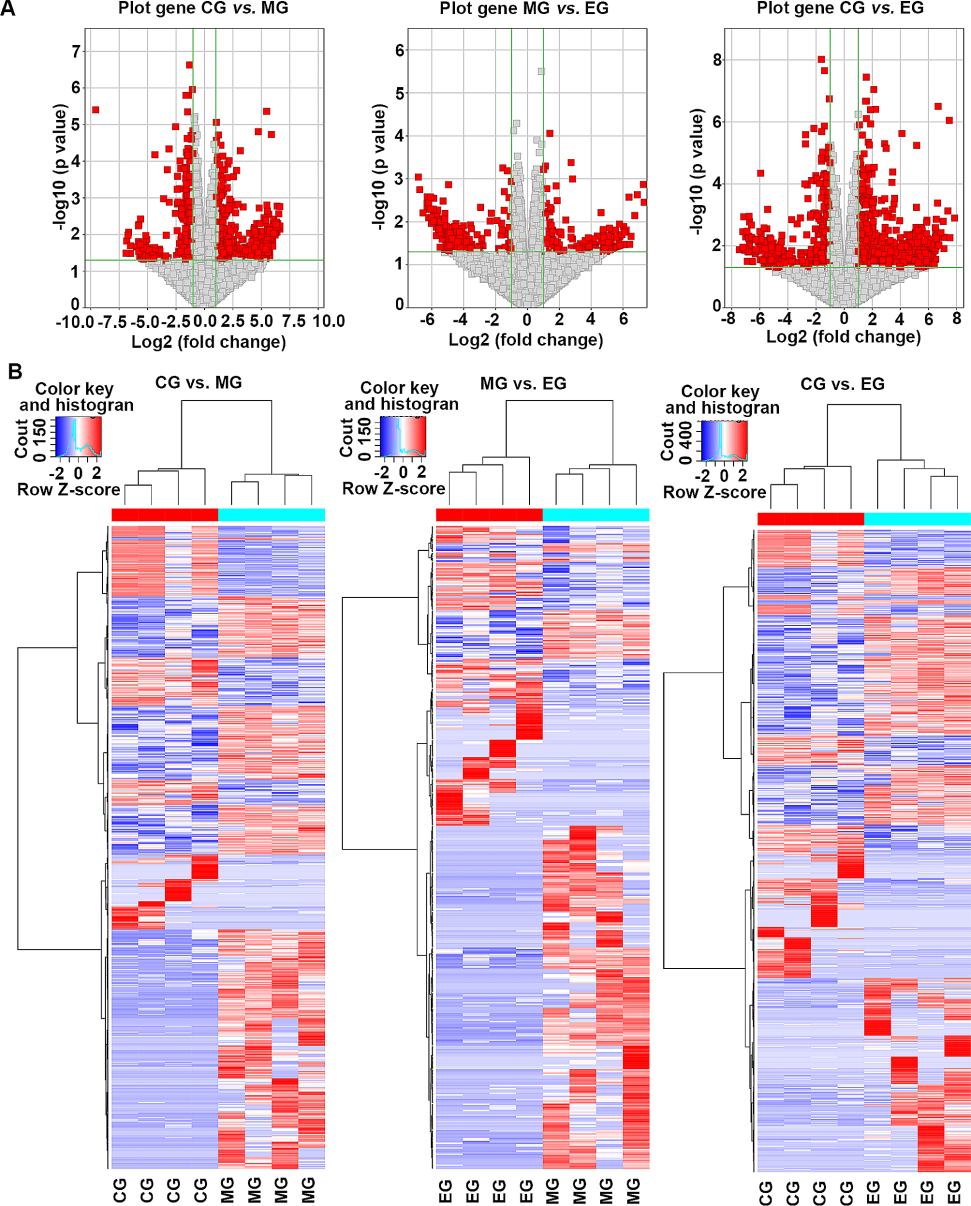
Correlation of DEGs among three different groups (**A**) and hierarchical clustering analysis of DEGs (**B**) in the lung tissues were conducted in the subsequent analysis. Volcano maps and heat maps revealed the significance of DEGs expression, and those DEGs with more significant differences in expression will be focused on. CG, control group; MG, model group; EG, exercise group

In the comparison of lung tissue genes among the three groups of mice, numerous DEGs were identified (Figs. 4 and 5). Specifically, 730 DEGs were found in the comparison between the CG and MG groups, with 484 upregulated and 246 downregulated genes. Similarly, 412 DEGs were identified in the comparison between the MG and EG groups, with 152 upregulated and 260 downregulated genes. Finally, the comparison between the CG and EG groups revealed 1034 DEGs, with 655 upregulated and 379 downregulated genes. Further analysis of DEGs among each group revealed that 61 genes were upregulated and 80 genes were downregulated following exercise intervention. However, exposure to cigarette smoke resulted in the upregulation of 249 genes and the downregulation of 121 genes. Notably, potassium voltage-gated channel subfamily E regulatory subunit 4 (Kcne4) was consistently downregulated in all three intergroup comparisons. Additionally, Cd200 receptor 2 (Cd200r2), lymphocyte antigen 6 family member I (Ly6i), and matrix metallopeptidase 12 (Mmp12) were found to be upregulated in all three intergroup comparisons. A total of 75 genes were found to be upregulated in the MG vs. CG comparison, while they were downregulated in the EG vs. MG comparison (as shown in Table I). Similarly, 19 genes were downregulated in the CG and MC comparison but upregulated in the EG and MG comparison (as shown in Table II). Notably, the family with sequence similarity 71 member A (Fam71a) and heat shock protein family A member 1B (Hspa1b) genes were found to be downregulated in both the CG and MG groups, but upregulated in the EG vs. MG and EG vs. CG comparisons.

**Table I:** Top 10 genes upregulated in MG vs. CG but downregulated in EG vs. MG, sorted by magnitude of Log-fold change

Gene ID	Symbol	Name	*P*-Value	Log FC
327,799	Usp44	Ubiquitin specific peptidase 44	0.0053	6.29
11,418	Asic2	Acid-sensing (proton-gated) ion channel 2	0.0051	6.09
23,925	Kel	Kell blood group	0.0071	6.08
14,761	Gpr27	G protein-coupled receptor 27	0.0037	6.08
100,038,683	Gm10775	Predicted gene 10,775	0.0051	6.04
74,176	Tgm5	Transglutaminase 5	0.0076	5.75
243,168	Hsd17b13	Hydroxysteroid (17-beta) dehydrogenase 13	0.0121	5.75
240,595	Kcnv2	Potassium channel, subfamily V, member 2	0.0277	5.72
629,147	Ctxn3	Cortexin 3	0.0178	5.68
436,240	Foxr2	Forkhead box R2	0.0105	5.67

*Note:* log FC, log (fold-change). The above DEGs were upregulated after smoke exposure but downregulated after exercise training, indicating that the effect of exercise training on lung tissue might be related to the above genes.

**Table II:** Top 10 genes downregulated in CG vs. MG but upregulated in EG vs. MG, sorted by magnitude of Log-fold change

Gene ID	Symbol	Name	*P*-Value	Log FC
18,509	Pax7	Paired box 7	0.0058	7.27
192,167	Nlgn1	Neuroligin 1	0.0137	6.89
14,402	Gabrb3	Gamma-aminobutyric acid (GABA) A receptor	0.0161	5.78
13,615	Edn2	Endothelin 2	0.0231	5.27
171,382	Trpm8	Transient receptor potential cation channel, subfamily M, member 8	0.0461	4.16
223,645	Mroh6	Maestro heat-like repeat family member 6	0.0283	3.65
20,339	Sele	Selectin, endothelial cell	0.0014	2.74
193,740	Hspa1a	Heat shock protein 1 A	0.0011	2.41
15,109	Hal	Histidine ammonia lyase	0.0288	1.88
20,302	Ccl3	Chemokine (C-C motif) ligand 3	0.0037	1.79

Abbreviation: logFC, log (fold-change). The above DEGs were downregulated after smoke exposure but upregulated after exercise training, indicating that the effect of exercise training on lung tissue might be related to the above upregulated genes.

### Molecular functions of DEGs

**Fig. 6:**
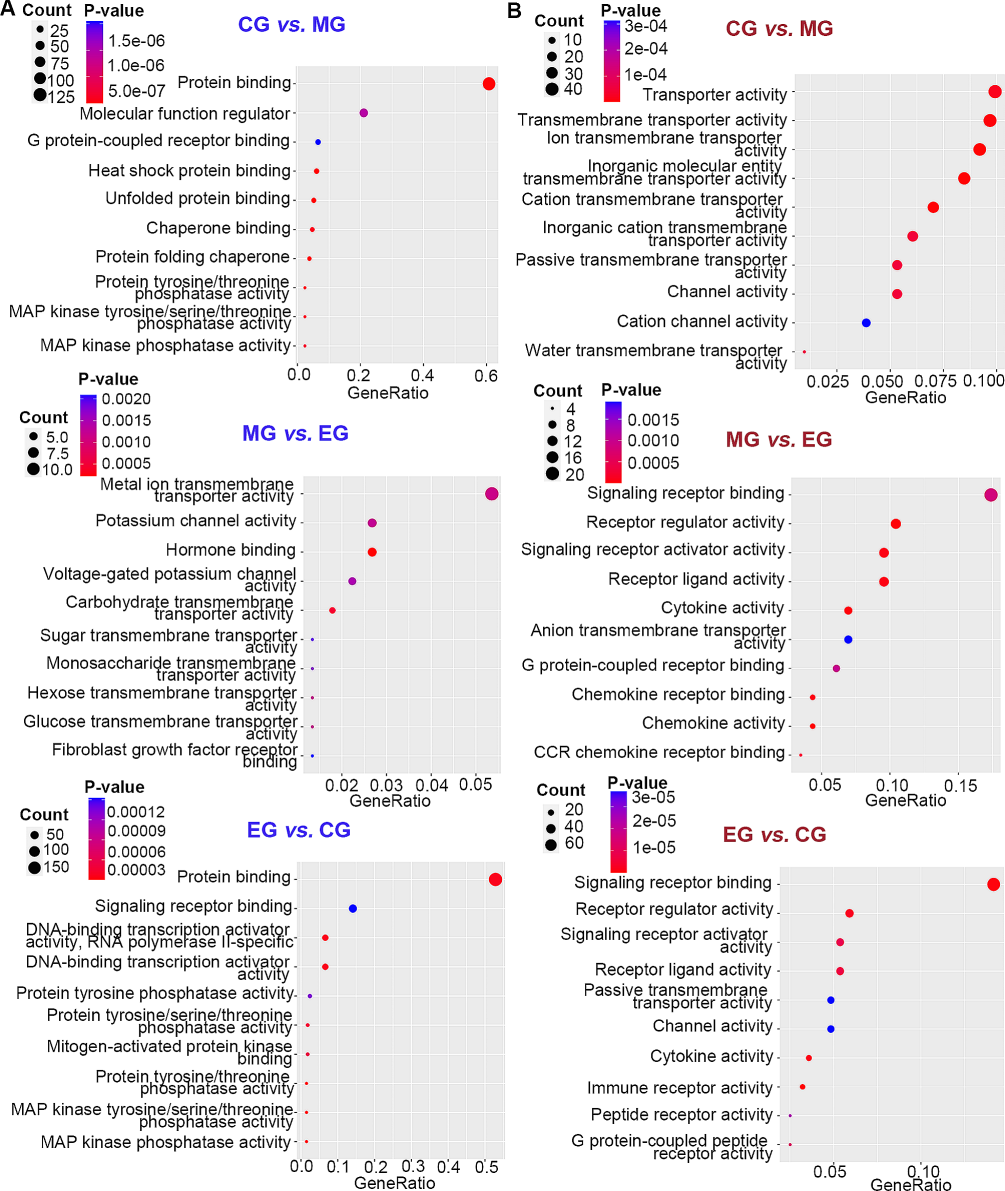
Molecular function of differentially expressed genes. (**A**) GO analysis of downregulated DEGs; (**B**) GO analysis of upregulated DEGs. The above results helped to understand the common characteristics of the molecular functions of the selected DEGs. According to the GeneRatio, those functions with more enriched DEGs and smaller P-values will be concerned. CG, control group; MG, model group; EG, exercise group

The molecular function aspect of the GO analysis was focused on investigating the molecular mechanism of exercise in improving pulmonary function in COPD **(**Fig. 6**)**. In the comparison of MG vs. CG, we observed that the downregulated DEGs were mainly concentrated in protein binding, such as G protein-coupled receptor binding, heat shock protein binding, unfolded protein binding and chaperone binding. Nevertheless, the upregulated DEGs were mainly related to transporter activity, including transmembrane transporter activity, ion transmembrane transporter activity, inorganic molecular entity transmembrane transporter activity, and cation transmembrane transporter activity. Moreover, the downregulated DEGs in the MG vs. EG comparison were mainly associated with metal ion transmembrane transporter activity, potassium channel activity, hormone binding and voltage-gated potassium channel activity. The upregulated DEGs were mainly related to signaling receptor binding, which included G protein-coupled receptor binding, chemokine receptor binding, and CCR chemokine receptor binding. Our findings also revealed that a substantial number of downregulated DEGs (EG vs. CG) were enriched in protein binding and signal receptor binding, with numerous DEGs associated with the mitogen-activated protein kinase (MAPK) signaling pathway. The DEGs that were upregulated in the EG vs. CG comparison were primarily involved in signaling receptor binding and receptor regulator activity.

### KEGG enrichment analysis

**Fig. 7:**
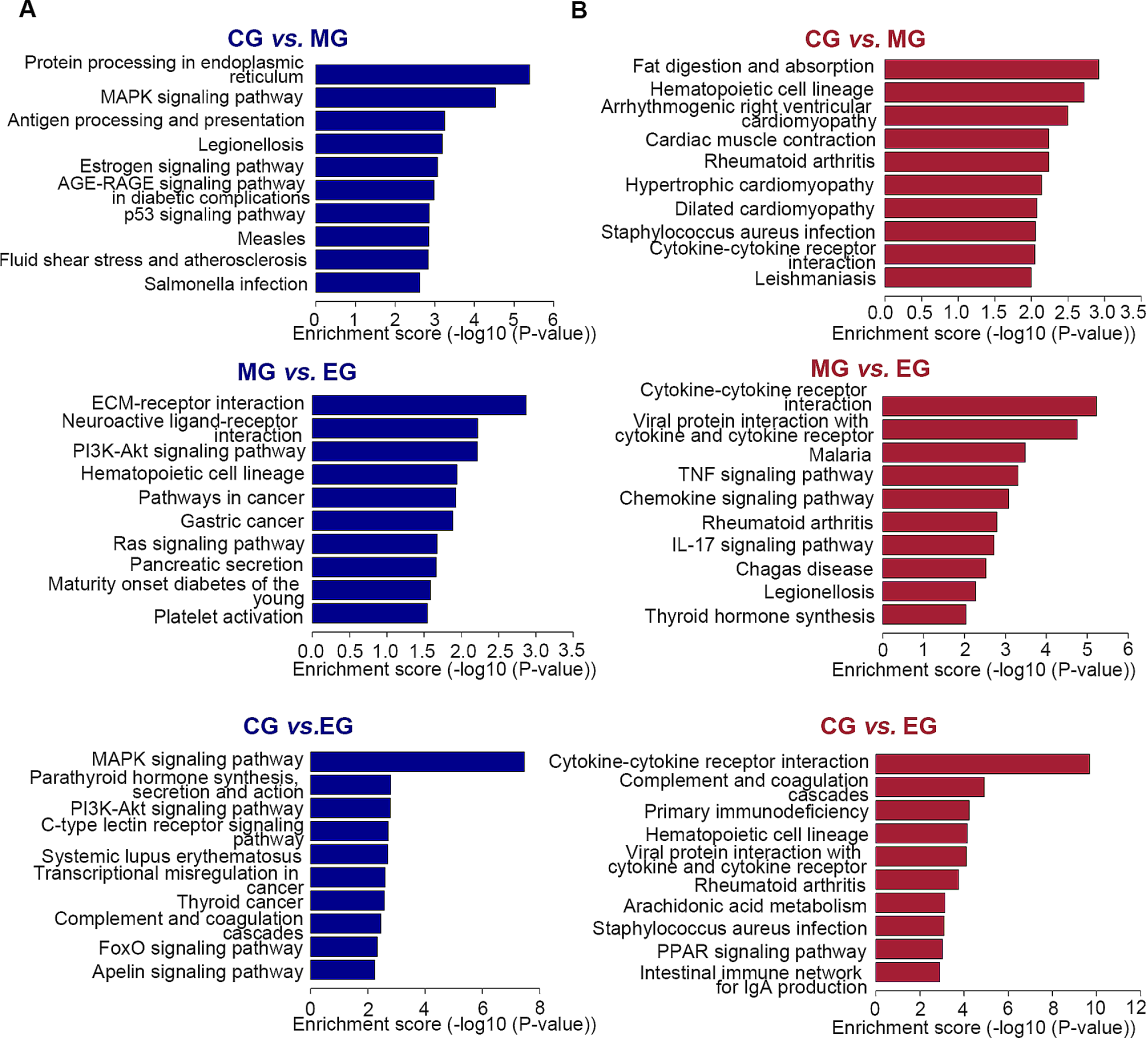
KEGG pathway analysis of DEGs. (**A**) KEGG pathway analysis of downregulated DEGs; (**B**) KEGG pathway analysis of upregulated DEGs. Signaling pathways with high enrichment score will be considered as potential mechanisms by which exercise improves respiratory function in COPD. CG, control group; MG, model group; EG, exercise group

Moreover, DEGs were identified by KEGG enrichment analysis to related pathways, and Fisher P value < 0.05 was used as a criterion to screen for significantly enriched pathways **(**Fig. 7**)**. After multiple recombination comparisons, the top 10 pathways were identified. Protein processing in the endoplasmic reticulum, MAPK signaling pathway, and antigen processing and presentation pathways were significantly inhibited in the MG and CG comparison. The pathways of fat digestion and absorption and cytokine-cytokine receptor interaction were significantly enhanced. The extracellular matrix (ECM)-receptor interaction, neuroactive ligand-receptor interaction, and phosphatidyl inositol 3 kinase - protein kinase B (PI3K-Akt) signaling pathway were significantly decreased in the comparison of EG and MG. Conversely, the cytokine-cytokine receptor interaction, tumor necrosis factor (TNF) signaling pathway, and chemokine signaling pathway were significantly enhanced. Additionally, in comparing EG and CG DEGs, we found that the MAPK signaling pathway, parathyroid hormone synthesis-secretion and action, PI3K-Akt signaling pathway, and C-type lectin receptor signaling pathway were significantly decreased. However, cytokine-cytokine receptor interactions, complement and coagulation cascades, and primary immunodeficiency were significantly enhanced.

## Discussion

The mechanism of exercise-related pulmonary rehabilitation is a crucial area of research, particularly as it is the only nonpharmaceutical approach currently available for improving respiratory dysfunction in COPD patients. In this study, a COPD mouse model was established through systemic CSE, and exercise intervention was subsequently implemented at an appropriate intensity. Transcriptomics was utilized as the primary method for this mechanistic exploration. This study aimed to investigate the potential role of exercise in improving pulmonary function and its impact on molecular transcription in lung tissue. The findings of this study may provide insights into the underlying mechanism of exercise-related pulmonary rehabilitation.

### Positive effects of exercise on the respiratory phenotype of COPD

Exercise training has been shown to have positive effects on respiratory function in both clinical and basic research studies. Specifically, a study conducted on stable COPD patients found that eight weeks of low-intensity exercise training at home significantly improved respiratory indicators [26], which were measured using the modified Medical Research Council dyspnea (mMRC) and COPD assessment test (CAT) scales. Another study indicated that engaging in aerobic exercise of an appropriate intensity improved pulmonary function indicators like FEV1 and FVC [27]. Notably, even low-intensity exercises like yoga and Tai Chi demonstrated positive effects on the predicted FEV1% of pulmonary function in COPD [10], emphasizing the beneficial impact of low-intensity aerobic exercise on pulmonary function in COPD improvement. Basic research has demonstrated that appropriate exercise training enhances respiratory function in COPD model animals [28]. Moreover, our previous research confirmed the beneficial effects of moderate-intensity water-based aerobic exercise on lung capacity and ventilation function in rats with COPD [29]. This study, despite its limitations, reinforced the notion that exercise enhanced pulmonary function in mice with COPD. The results served as a basis for further investigation into the underlying mechanisms responsible for this improvement.

The results of staining lung tissue, based on the confirmation of the successful establishment of a COPD model, revealed potential extrapulmonary pathways for exercise to improve pulmonary function in COPD. In MG mice exposed to cigarette smoke for six months, the emphysema symptoms were similar to those observed in previous studies [30, 31], including thickening of the airway epithelium and smooth muscle, infiltration of inflammatory cells, destruction of alveolar structure, and widening of alveolar septa. The observed symptoms confirmed the successful establishment of the COPD animal model in our study. Additionally, we compared the cross-sectional area of mouse alveoli and found that exercise training did not have a significant impact on the alveolar fusion phenomenon in mice with COPD. However, previous research indicated that exercise intervention promoted airway remodeling by impacting airway inflammation and limiting the overgrowth of smooth muscle [32]. Moreover, numerous studies have demonstrated that exercise training enhances respiratory function in individuals with COPD by activating respiratory muscle function and preventing respiratory muscle atrophy [33–35].

### Potential gene targets for pulmonary rehabilitation

The DEGs that were upregulated in the MG vs. CG comparison but downregulated in the EG vs. MG comparison, or downregulated in the MG vs. CG comparison but upregulated in the EG vs. MG comparison, were of particular interest to us. This finding helps to explain the potential mechanisms by which exercise improves pulmonary function in COPD through the dynamic expression of genes.

Exposure to smoke leads to the up-regulation of a significant number of genes, whereas exercise intervention results in their suppression. Among the ubiquitin-specific protease (USP) family, USP 44 plays a crucial role in regulating various mechanisms by targeting multiple downstream factors through its deubiquitinating activity [36]. USP44 has been shown to play a role in immune regulation and stabilization of immune function in inflammatory disease models through fork head box protein 3 (FOXP3). This molecule has also been found to regulate autophagy by controlling the expression of histone H2B monoubiquitination (H2Bub1) [37]. Although there is no current data supporting the involvement of USP44 in the pathogenesis of COPD or the effect of exercise on COPD, the role of USPs in COPD-related skeletal muscle dysfunction has been preliminarily confirmed [38]. The findings of this study indicated that USP44 was upregulated in the COPD mouse model and downregulated after exercise intervention. This result suggested that USPs, represented by USP44, might have a role in exercise-related pulmonary rehabilitation, warranting further investigation. Additionally, hydroxysteroid (17- beta) dehydrogenase 13 (Hsd17b13) is a key member of the 17β-hydroxysteroid dehydrogenase (HSD17B) family and is highly expressed in nonalcoholic fatty liver disease. This molecule is primarily expressed in the liver, while expression in the lungs is low [39]. The biological function of Hsd17b13 is closely related to the regulation of the occurrence, growth, and degradation of lipid droplets in the liver. Currently, lipid metabolism in COPD patients has garnered increasing attention from scholars, with a focus on factors related to fat, such as chemerin [40]. However, research has also identified several DEGs, including acid-sensing ion channel 2 (Asic2), transglutaminase 5 (Tgm5), and forkhead box R2 (Foxr2), whose biological functions were not closely associated with existing COPD-related factors.

Interestingly, exercise training activated another group of the genes that exhibited the opposite expression trend, and these genes were downregulated after exposure to cigarette smoke. Paired box 7 (Pax7), among these genes, is a crucial member of the Pax transcription factor family that plays an important role in the development of various tissues. This molecule is highly expressed in the central nervous system and skeletal muscle but is expressed at low levels in lung tissue. Pax7 plays a major role in adult growth and muscle regeneration after birth [41]. Recent research found that the expression of Pax7 was significantly insufficient in the satellite cells of the lateral muscles of COPD patients with muscle loss [42]. Exercise training is an effective strategy to prevent muscle loss by increasing Pax7 expression. Our study’s findings also indicated that certain central nervous system genes, such as neuroligin 1 (Nlgn1) and gamma-aminobutyric acid A receptor beta 3 (Gabrb3), exhibited significant expression in COPD. These genes, along with related genes, have been linked to various biological effects [43, 44]. As a result, we speculate that their presence may have a substantial impact on the physical and cognitive health of individuals with COPD [45]. The involvement of transient receptor potential (TRP) channels in regulating physical and chemical stresses in the respiratory tract has recently attracted increased attention [46]. Evidence suggests that the TRP Member 8 (TRPM8) subtype channel is linked to cough and airway constriction caused by inhaling cold air [47]. Additionally, published data indicated that the activation of TRPM8 channels might result in the production of inflammatory mediators [48]. There is still some controversy over the current data, as some studies have suggested that the activation of TRPM8 channels inhibits the cough response [49, 50]. However, the results of this study indicated that TRPM8, as a differentially expressed gene, was downregulated after exposure to cigarette smoke but activated after exercise training, which suggested a positive role for TRPM8 in improving pulmonary function in mice with COPD through exercise. Further research is needed to determine the exact role of TRPM8 in exercise-related lung rehabilitation. Notably, this study also highlighted the potential benefits of the heat shock protein family A protein 1 A (Hspa1a) in improving pulmonary function through exercise. Hspa1a is induced under stress conditions as a cellular defense mechanism, and since the airway of individuals with COPD is under such stress conditions, Hspa1a could be a valuable target for future research in this area. According to previous research, Hspa1a is thought to combat the detrimental effects of oxidative stress and regulate inflammation [51]. Based on the findings of this study, the potential of Hspa1a to enhance respiratory function in patients with COPD through exercise warrants further investigation. This research could lead to the identification of Hspa1a as a key player in the molecular mechanisms of exercise-induced lung rehabilitation. Moreover, exercise training significantly upregulated chemokine factors represented by C-C motif chemokine ligand 3 (Ccl3), which may be associated with the cascade reaction resulting from immune system activation after exercise [52, 53]. The immune response produced by the body after exercise is complex.

In the intergroup comparison, some genes showed sustained down-regulation or up-regulation, which aroused our concern. The Kcne4 protein serves as an auxiliary subunit of the voltage-gated potassium channel (Kv1.3), which is a single transmembrane channel. It plays a role in regulating the potassium ion current and controlling the abundance of the channel on the cell surface and has multiple physiological functions [54]. In response to alveolar hypoxia, mitochondrial sensors dynamically modify reactive oxygen species and redox pairs in pulmonary artery smooth muscle cells (PASMC). As a result, potassium channels are inhibited, leading to the depolarization of PASMC. This depolarization activates voltage-gated calcium channels and increases the cellular solute calcium, causing vasoconstriction. Alveolar hypoxia leads to the contraction of internal pulmonary arteries, redirecting blood flow to lung segments with better oxygen supply. This optimization helps in achieving ventilation/perfusion matching and systemic oxygen supply [55]. The continuous decrease in Kcne4 gene expression in the lungs of MG mice may be associated with this phenomenon. The reduced transcription level of Kcne4 after exercise may also be linked to the regulation of pulmonary blood metabolism in a state of low voltage-dependent potassium channel activation [56]. Additionally, Kv channels have been suggested to play a significant role in the activation and proliferation of leukocytes, with Kcne4 acting as an inhibitory Kv1.3 chaperone in leukocytes [57]. The downregulation of Kcne4 may also be a potential target for exercise-induced regulation of the body’s immune response. CD200R2 is a transmembrane glycoprotein located on the cell surface and acts as a receptor for leukocyte differentiation antigen CD200. The interaction between CD200 and CD200R plays a crucial role in the immune system of individuals with COPD. Studies have shown that increased expression of CD200 promotes the adhesion between T cells and endothelial cells, stimulates the secretion of anti-inflammatory factors, and inhibits inflammatory responses [58, 59]. Ly6i, also known as lymphocyte antigen-6/urokinase-typeplasminogen activator receptor, belongs to the superfamily and is highly expressed in monocytes and neutrophils [60, 61]. While the specific biological role of Ly6i is still unclear, it is involved in various cellular functions such as leukocyte differentiation, cell adhesion, cell migration, and cytokine production [60]. Moreover, Ly6i has close associations with inflammatory diseases. The continuous up-regulation in CD200R2 and Ly6i levels may indicate a response of the body and exercise training to airway inflammation, although further evidence is required to support this hypothesis. Mmp12 has been shown to degrade ECM and participate in the pathogenesis of COPD, and the expression level of Mmp12 in macrophages of COPD patients is increased [62, 63]. Furthermore, Mmp12 activates protease-activated receptor-1, upregulates placenta growth factor, and leads to pulmonary emphysema [64]. Consistent with the above studies, Mmp12 was upregulated in lung tissue of mice pretreated with smoke exposure. However, we are not yet able to explain why Mmp12 transcription levels continue to rise after exercise, considering that previous study have demonstrated the inhibitory effect of exercise on Mmp12 [65]. Whether more complex regulatory mechanisms are involved remains to be explored.

### Potential pathways of pulmonary rehabilitation

**Fig. 8:**
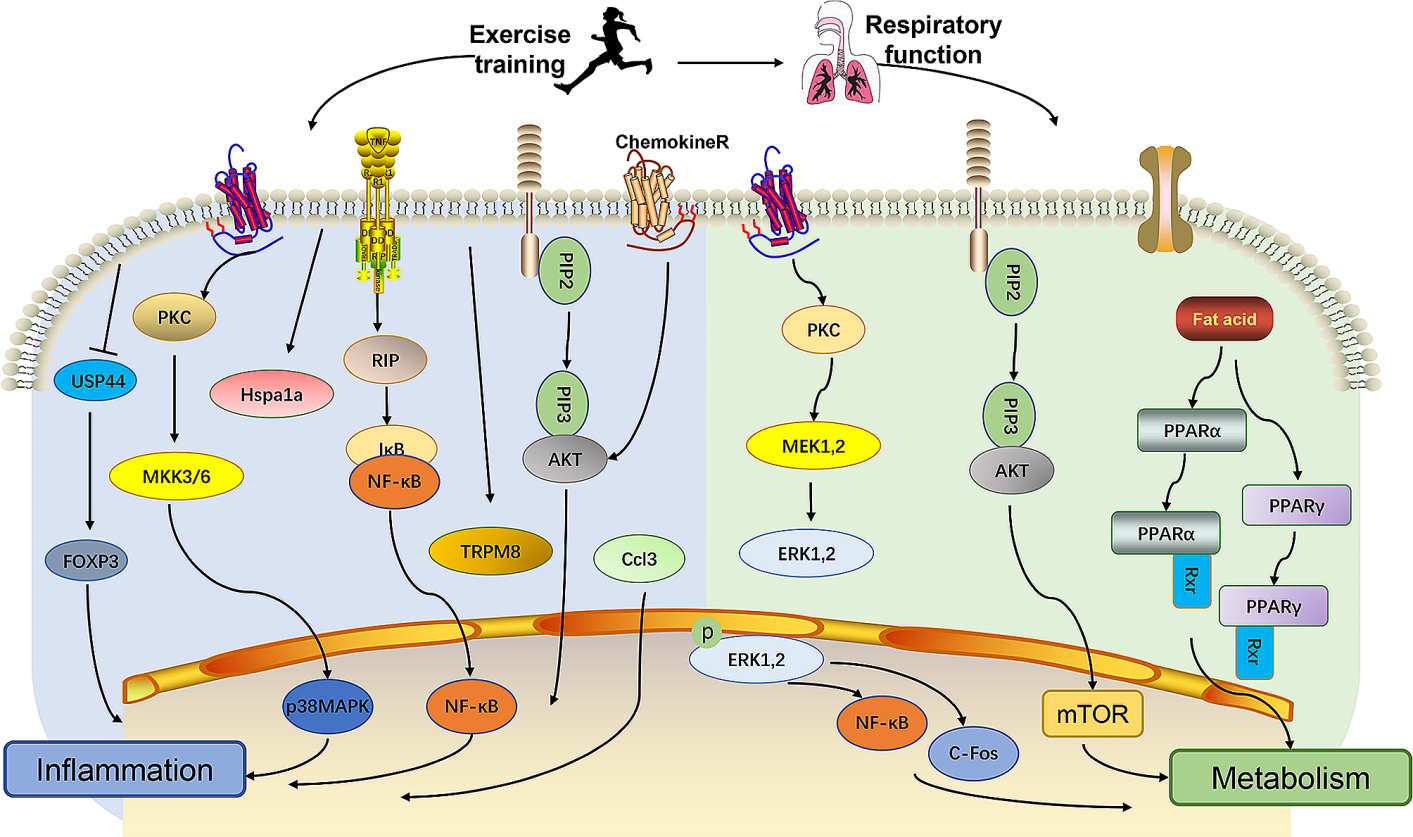
The potential pathways of exercise training to improve pulmonary function. Aerobic exercise may improve COPD respiratory dysfunction by regulating the body’s inflammatory response and metabolism. Multiple pathways, including MAPK signaling pathway, p53 signaling pathway, and PI3K-Akt signaling pathway, might be involved

Exercise training is a multitarget intervention that has been shown to have rehabilitative effects on various diseases and functional disorders. However, due to its complex nature, the specific mechanisms underlying exercise rehabilitation are difficult to determine. This study aimed to identify potential signaling pathways by which exercise improves pulmonary function in mice with COPD by analyzing numerous DEGs. Through screening and enrichment, numerous signaling pathways were identified, all of which have the potential to contribute to exercise-induced improvements in pulmonary function. After analyzing the relevant signaling pathways, we concluded that exercise training might primarily regulate inflammation and metabolism **(**Fig. 8**)**, which led to its positive-functional effects.

Several enriched pathways, including cytokine-cytokine receptor interaction, TNF signaling pathway, chemokine signaling pathway, and interleukin (IL)-17 signaling pathway, are directly related to the inflammatory response and serve as direct transmitters of inflammatory signals. Additionally, other relevant signals, such as the MAPK signaling pathway, tumor protein 53 (p53) signaling pathway, and PI3K-Akt signaling pathway, play a role in regulating the inflammatory response. We found that exercise training has a significant impact on airway inflammation in mice with COPD, which is consistent with previous studies [53]. Clinical trials demonstrated that both moderate-intensity cycling [66] and treadmill training [67] could lower blood inflammation levels in patients with COPD. Furthermore, recent research has indicated that regular and low-moderate treadmill training protects against smoking-induced lung disease by exerting anti-inflammatory effects. According to experimental animal studies, treadmill training has been found to reduce lung inflammation [68, 69] and remodeling [70] after exposure to cigarette smoke or induced asthma. In particular, treadmill training has been shown to increase the Th1 response and suppress Th2 cytokine levels in the lungs of mice that smoke. Additionally, exercise has been found to enhance antioxidant defenses and reduce markers of oxidative stress [71]. The positive effects of exercise on lung rehabilitation have been demonstrated in mice exposed to cigarette smoke. Specifically, exercise preconditioning was found to significantly reduce bronchoalveolar capillary permeability, inflammatory cell infiltration, epithelial thickening, expression of proliferating cell nuclear antigen, mucin 2, cytokines, chemokines, adhesion molecules, and activation of nuclear factor kappa B (NF-κB) [72]. Hence, further investigation of inflammatory signaling pathways is crucial to fully understand these effects.

There are numerous signaling pathways that contribute to the therapeutic mechanism of exercise in lung rehabilitation, with many identifying its effects on body metabolism. Multiple examples include the MAPK, fat digestion and absorption, pancreatic secretion, p53, PI3K-Akt, and peroxisome proliferators activated recepotor (PPAR) signaling pathways. These pathways impact body metabolism in various ways and are all potentially relevant to the benefits of exercise in lung rehabilitation. A clinical study demonstrated that individuals with COPD were at a higher risk of developing metabolic disorders compared to those who were healthy [73]. This result might be attributed to several factors, such as chronic inflammatory response, oxidative stress, abnormal immune metabolism, and corticosteroid therapy in COPD patients [74, 75]. The positive effects of exercise training on metabolism in COPD have been confirmed in multiple studies [76, 77], and these effects were closely related to the Akt [78] and MAPK signals [79]. Exercise training has the potential to improve the metabolic level of the body and activate various signals to correct imbalances, ultimately leading to rehabilitation. Adipokines, which have the potential to influence both the inflammatory response and insulin levels, have attracted increased attention [40].

Although multiple DEGs and enriched pathways were identified in this study, notably, these findings were not validated or further explored, which represents a major drawback of the study. In future research, we intend to delve deeper into the role of key genes, such as USP 44, Hsd17b13, and Hspa1a, as well as the associated pathways, to investigate their potential in improving pulmonary function in COPD through exercise. By addressing this gap, we hope to generate valuable insights for subsequent studies and encourage more scholars to participate in the field of sports-related lung rehabilitation.

## Conclusions

Collectively, transcriptomics analysis was performed on lung tissues from three groups of mice to identify potential mechanisms involved in the exercise-induced improvement of COPD-related respiratory dysfunction in our study. Our results suggested that exercise might modulate genes and pathways associated with inflammation and metabolism. However, further research is needed to establish a causal relationship between exercise and COPD-related respiratory dysfunction. Further research is necessary to determine the causal relationship between these biomarkers and improvements in the COPD phenotype. Therefore, conducting follow-up verification of the aforementioned biomarkers and biological signals is essential for exploring the molecular biological mechanism of exercise-dependent pulmonary rehabilitation.

## Data Availability

The datasets generated and analyzed during the current study are available in the SRA database (PRJNA976479), https://www.ncbi.nlm.nih.gov/sra/PRJNA976479.
